# Successful Conservative Treatment of an Elderly Patient with Corrosive Proctocolitis

**DOI:** 10.1155/2019/9135378

**Published:** 2019-12-06

**Authors:** Chalerm Eurboonyanun, Somchai Ruangwannasak, Kulyada Eurboonyanun, Anan Sripanuskul

**Affiliations:** ^1^Department of Surgery, Faculty of Medicine, Khon Kaen University, Thailand; ^2^Department of Radiology, Faculty of Medicine, Khon Kaen University, Thailand

## Abstract

Corrosive proctocolitis has occurred after accidental contamination of endoscopes in most patients. But accidental administration of corrosive agents for bowel cleansing can occur. The agents implicated for chemical colitis is 15% hydrochloric acid and 2% ethoxylated alcohol. We present a case of corrosive proctocolitis, present with anal pain and bloody diarrhea. Endoscopy revealed edema, erythema, and friability of the colonic mucosa. An experience of successful nonoperative treatments has been demonstrated.

## 1. Introduction

Corrosive proctocolitis has previously occurred by contamination of endoscopic cleansing liquid [1–3]. Some enema, such as soap and detergent, can also cause proctocolitis [4, 5]. However, accidental administration of toilet cleaning agents rarely occurs. We describe one such unusual case of caustic colitis that was the encounter in this situation.

## 2. Ethical Consideration

This retrospective case report was approved by the Ethics Committee for Human Research based on the Declaration of Helsinki and the ICH good clinical practice guidelines. Clinical data were obtained by reviewing medical records.

## 3. Case Presentation

A Thai 80-year-old male was admitted to the hospital with a history of anal pain and bloody diarrhea without abdominal pain. His symptoms occurred five hours after accidental enema administration to relive constipation with liquid toilet cleaners (15% hydrochloric and 2% ethoxylated alcohol, pH 0.5-1.0).

A physical examination revealed mild anal pain without abdominal pain nor guarding. Blood test showed a hemoglobin of 13.1 g/dL, white blood cell count of 17,900/*μ*L, and platelet count of 218,000/*μ*L.

**Figure 1:**
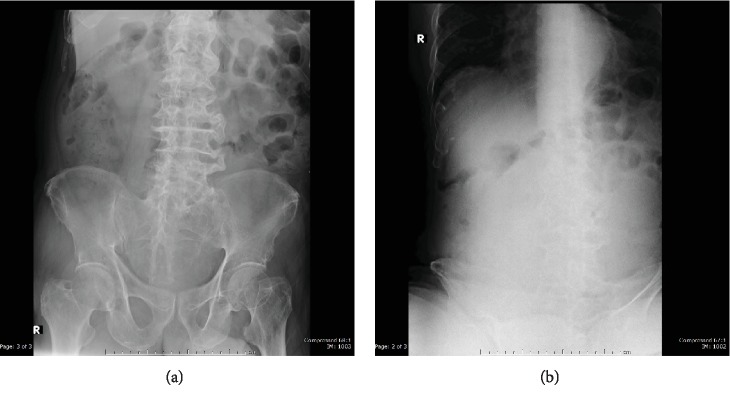
(a, b) Supine and upright abdominal radiographs show decreased amount of bowel gas in the lower abdomen/pelvic cavity.

Acute abdominal film (Figures 1(a) and 1(b)) shows a decreased amount of bowel gas in the lower abdomen/pelvic cavity.

**Figure 2:**
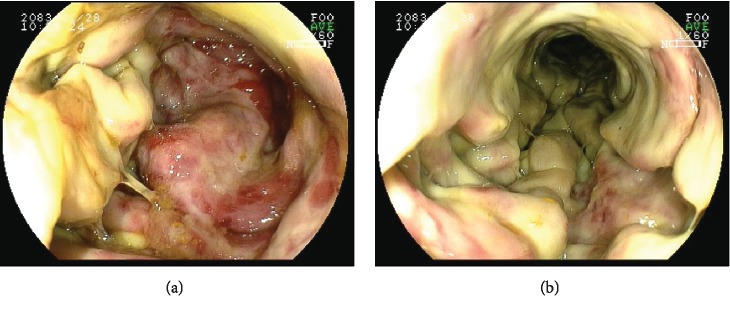
(a, b) Circumferential friability, whitish membrane, edema, erythema, and superficial ulceration of mucosa from the anus to the sigmoid colon.

The patient was diagnosed with corrosive proctocolitis. He was conservatively treated with intravenous antibiotics and fluid replacement therapy, restricting the oral intake of food/liquids for 2 days before elective colonoscopy. Colonoscopy showed circumferential friability, whitish membrane, edema, erythema, and superficial ulceration of mucosa from the anus to the sigmoid colon (Figures 2(a) and 2(b)).

After colonoscopy, the patient was able to consume a soft diet orally without abdominal discomfort. His bleeding and pain had also improved. He was discharged on the 5^th^ day after admission.

**Figure 3:**
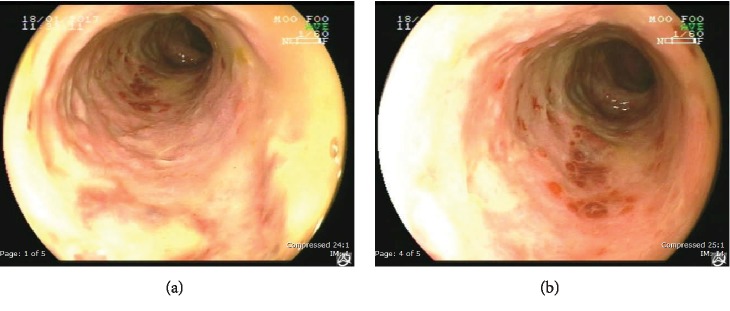
(a, b) Slightly pale mucosa with focal area of erythema from the anus to the sigmoid colon with narrowing of the caliber of the sigmoid colon.

Six weeks after, repeated colonoscopy showed whitish membrane, erythema, and stricture from the anus to the sigmoid colon (Figures 3(a) and 3(b)). One year after, he still had constipation, but he denied any further investigation or surgery. He had used saline enema to relieve constipation.

## 4. Discussion

Chemical proctocolitis is less common compared to chemical esophagitis for suicidal intent in adults. Chemical colitis has been reported to occur after the rectal administration of various chemicals including alcohol, detergent, hydrogen peroxide, glutaraldehyde, herbal medicine, and strong acids/base. One of the most common causes is residual glutaraldehyde on the surface of the endoscope after the cleaning procedure [6]. Hydrogen peroxide is also a common cause of chemical colitis, which some people believe to be a home remedy for constipation in children [7, 8]. Most chemical proctocolitis patients were successfully treated with a conservative strategy with bowel rest and parenteral hydration. But there is no management guideline following endoscopic findings as corrosive esophagitis described by Zagar et al. to predict outcomes [9].

In this case, chemical proctocolitis is caused by very rare agents such as the toilet cleaner. An experience of successful nonoperative treatments in the modified Zagar classification grade IIB had been demonstrated. Although the colonic stricture occurred, there was no need for any other operation.

In conclusion, the patient who presented with corrosive proctocolitis was able to be treated conservatively in the acute phase. However, colonic stricture can occur in the late phase, but any other procedures to reduce late complications require further investigation.
